# Complete and Circularized Genome Assembly of a Human Isolate of Vibrio navarrensis Biotype *pommerensis* with MiSeq and MinION Sequence Data

**DOI:** 10.1128/MRA.01435-20

**Published:** 2021-02-04

**Authors:** Keike Schwartz, Maria Borowiak, Carlus Deneke, Veronika Balau, Claudia Metelmann, Eckhard Strauch

**Affiliations:** a German Federal Institute for Risk Assessment, Department of Biological Safety, Berlin, Germany; b Institut für Medizinische Diagnostik Labor Greifswald, Greifswald, Germany; Georgia Institute of Technology

## Abstract

Vibrio navarrensis is a rare human pathogen. Strains of Vibrio navarrensis biotype *pommerensis* were isolated from seawater of the Baltic Sea. Recently, a strain of this biotype was recovered from a human patient. The isolate contains two circular chromosomes and a large plasmid with a size of 180 kb.

## ANNOUNCEMENT

Vibrio navarrensis was found in river water and sewage in Spain (1). Later, isolates obtained from clinical samples suggested a human-pathogenic potential for this species (2). V. navarrensis biotype *pommerensis* strains were detected in seawater samples from the Baltic Sea (3). Isolates of the biotype *pommerensis* revealed major differences in biochemical profiles as well as borderline values in DNA-DNA hybridization experiments, compared to the V. navarrensis reference strain ATCC 51183 (4).

Strains of the biotype *pommerensis* have rarely been discovered in German coastal waters (5); however, in summer 2020, a biotype *pommerensis* isolate was recovered from a feverish patient suffering from an erysipelas-like infection on one leg. Prior to infection, the patient had been bathing in the Baltic Sea. The new isolate (20-VB00237) was subjected to whole-genome sequencing for further investigation.

The isolate was obtained from a blood culture (BD Bactec Plus Aerobic/Anaerobic-F) using Columbia agar with 5% sheep blood according to the manufacturer’s recommendations (BD, Heidelberg, Germany). Subsequently, the isolate was cultivated for 24 h at 37°C in lysogeny broth. Genomic DNA was extracted using the PureLink genomic DNA minikit (Thermo Fisher Scientific, Waltham, MA, USA) and sequenced using MiSeq (Illumina, San Diego, CA, USA) and MinION (Oxford Nanopore Technologies [ONT], Oxford, UK) devices. An Illumina sequencing library was prepared using the Nextera DNA Flex kit. Paired-end sequencing was performed in 2 × 151-bp cycles on an Illumina MiSeq instrument using the MiSeq reagent kit v3 (600 cycles). Trimming of short reads using fastp v0.19.5 (6) resulted in 1.2 million high-quality reads (97.8% with a quality score of ≥Q30). An ONT sequencing library was prepared using the rapid barcoding kit and sequenced on an ONT MinION sequencer connected to an ONT MinIT v19.12.5 device (including Guppy base caller v3.2.10) using a FLO-MIN106 R9 flow cell. The reads obtained were trimmed using Porechop v0.2.3 (https://github.com/rrwick/Porechop), filtered using NanoFilt v2.7.1 (7), and quality checked using NanoStat v1.4.0 (7). In total, 25,772 reads with a read *N*_50_ value of 12,481 bp and a mean read quality score of 10.9 were available.

The two data sets were assembled and circularized using Unicycler v0.4.8 including Pilon (8–10). Default parameters were used for all software unless otherwise noted. The assembly resulted in two circular bacterial chromosomes, i.e., chromosome 1 (3,534,271 bp) and chromosome 2 (1,395,396 bp), and a closed plasmid (pVN20-VB00237) of 180,139 bp. The overall G+C content of the bacterial genome was 47.9%. The genome sequence was annotated using PGAP v4.11 (https://www.ncbi.nlm.nih.gov/genome/annotation_prok). Results revealed the presence of 4,662 coding sequences (CDSs) (4,511 protein-coding CDSs and 151 pseudogenes) and 150 RNA genes (34 rRNAs, 112 tRNAs, and 4 noncoding RNAs).

**FIG 1:**
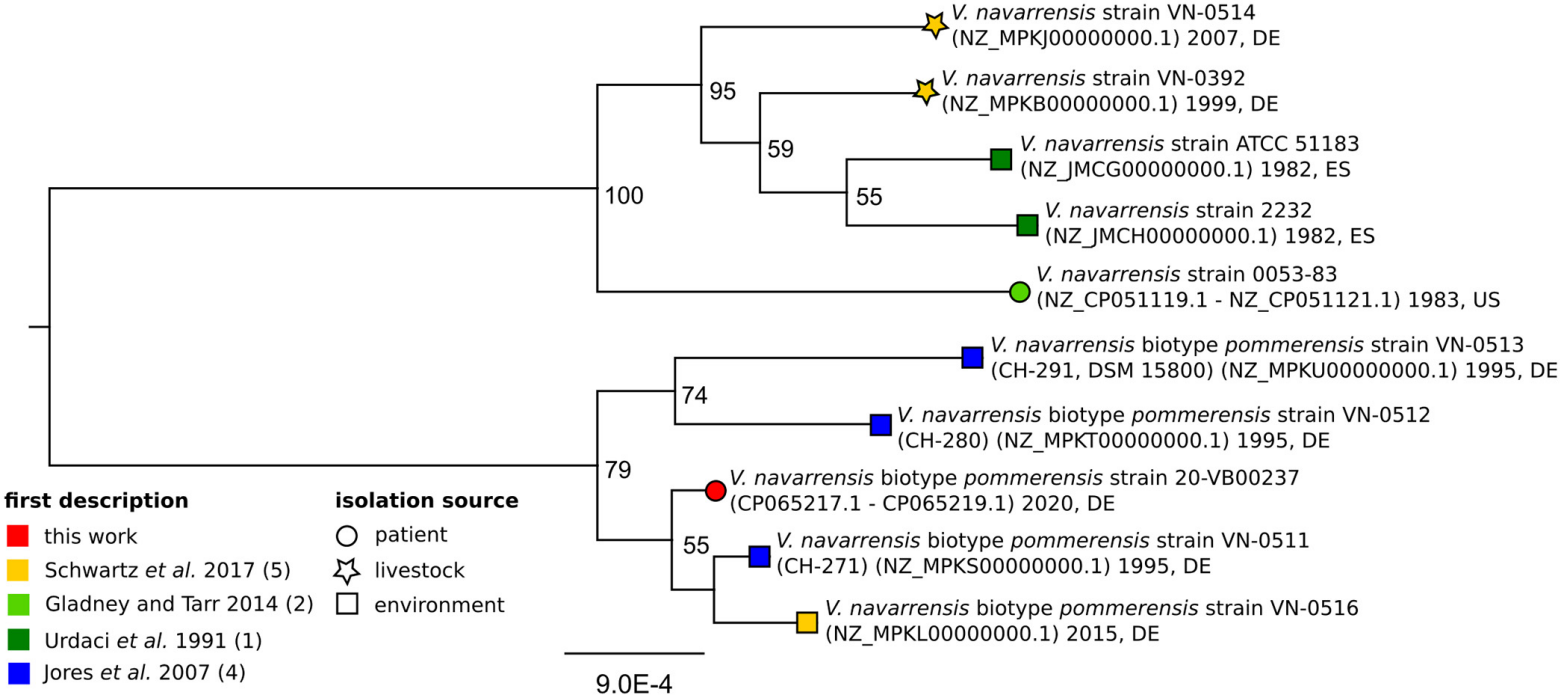
Best-scoring maximum likelihood tree based on a comparison of the amino acid sequences of 107 essential single-copy core genes of V. navarrensis biotype *pommerensis* strains and other published V. navarrensis strains using bcgTree. Numbers at the nodes designate bootstrap support values resulting from 100 bootstrap replicates. Numbers in parentheses are GenBank accession numbers. Dates in the node labels indicate the year of isolation, and abbreviations indicate the country of isolation (DE, Germany; ES, Spain; US, United States).

The isolate 20-VB00237 genome was compared to previously published V. navarrensis and V. navarrensis biotype *pommerensis* genomes. Therefore, all genomes were annotated using Prokka v1.1.3 (https://github.com/tseemann/prokka). Subsequently, phylogeny was inferred using bcgTree v1.1.0 (11), which compares the amino acid sequences of 107 single-copy core genes. The resulting maximum likelihood tree was visualized in Geneious v2020.2.2 (Biomatters, Auckland, New Zealand), manually rooted using the V. navarrensis node, and finalized using Inkscape. The final tree (Fig. 1) reveals that strain 20-VB00237 groups within a distantly related cluster of other V. navarrensis biotype *pommerensis* strains that were previously isolated from the Baltic Sea.

### Data availability.

The complete genome sequence of 20-VB00237 is available at NCBI (GenBank accession numbers CP065217 [chromosome 1], CP065218 [chromosome 2], and CP065219 [pVN20-VB00237]). Sequencing raw reads were deposited in the NCBI Sequence Read Archive (SRA) (accession numbers SRX9566546 [ONT data] and SRX9566545 [Illumina data]).
